# Pattern of anti-diabetic drugs prescribed in a tertiary care hospital of Bangladesh

**DOI:** 10.18203/2319-2003.ijbcp20160079

**Published:** 2016-01-28

**Authors:** Zuhayer Ahmed, M. A. Hafez, M. A. Bari, Jesmin Akhter

**Affiliations:** 1 Global Alliance for Vaccines & Immunization (Gavi), Faridpur, Bangladesh; 2 Department of Biostatistics, Bangladesh University of Health Sciences, Dhaka, Bangladesh; 3 Department Public Health, ASA University Bangladesh, Dhaka, Bangladesh

**Keywords:** Drug Utilization, Hypoglycemic agents, Tertiary healthcare

## Abstract

**Background:**

Globally, diabetes mellitus is a common endocrine disorder. This study was conducted for collecting the demographic details of diabetic patients and determining the pattern of drugs prescribed among them in outpatient department of a tertiary healthcare center.

**Methods:**

A descriptive type of cross-sectional study was carried out at the outpatient department of Endocrinology, Dhaka Medical College Hospital, Bangladesh from 1 May to 31 July, 2015. Diabetic patients receiving the management for at least 6 months were enrolled and interviewed by the researchers after getting informed written consent. Structured case record form was used for demographic data & prescription details. Data were analysed using computer in SPSS 22 and Microsoft Excel 2010.

**Results:**

Altogether 105 patients, 40 males (38.1%) and 65 females (61.9%) were enrolled with urban predominance (69.5%) where 51 (48.6%) were in the age group 47-61 years with a mean of 53.4 (SD±10.6) years. 70 (66.7%) had diabetic history of less than 5 years and 66 (62.9%) had at least one concurrent illness. Hypertension accounted for majority (34.3%) of complications. On an average, 5.62 (SD±3.16) drugs were advised per prescription for diabetes as well as associated co-morbidities and majority (23.8%) had 4 drugs. The majority of drugs (74.3%) were from local manufacturers. Most patients (62.9%) were prescribed with oral drugs singly. Metformin alone predominated in 41% prescriptions followed by the combination of Metformin and Sitagliptin (31.4%).

**Conclusions:**

The findings can serve as a guide to choose the formulation and combination of anti-diabetic drugs in this part of the world before developing & marketing any new drug.

## INTRODUCTION

Diabetes Mellitus (DM) is a common and very prevalent disease affecting about 25% of world population of both developed and developing countries.^1,2^ The number of people developing DM is currently increasing worldwide in an alarming exponential proportions; this may be connected to a rapid rise in risky health behaviors, urbanization and aging. There is an estimate of over 200 million people with diabetes in the world, 80% of whom reside in developing countries and is expected to be the most prevalent non-communicable disease by 2025.^2,3^ A recent meta-analysis showed that the prevalence of diabetes among adults had increased substantially, from 4% in 1995-2000 and 5% in 2001-2005 to 9% in 2006-2010 periods in Bangladesh.^4^ This country had a population of 149.8 million in 2011.^5^ According to the International Diabetes Federation, the prevalence will be 13% by 2030.^6^ There is paucity of data regarding drug use pattern in diabetic patients in Bangladesh. Study related to drug utilization of anti-diabetic drugs is of paramount importance to promote rational drug use and disseminate valuable information to health teams to ensure that. We can also say that irrational prescribing can lead to increased cost of drugs, which often leads to non-adherence.^7^

A study from the United States of America (USA) reported that about 1.3 million adults with disabilities did not take their medications as prescribed because of cost and as a result, more than half reported health problems later.^8^ For better control of DM with minimal complications of drugs, this information has no alternatives. A study on drug utilization can provide valuable information to the physicians, researchers, policy makers and the drug & therapeutics committee members to determine the drug use pattern.^7^

This study is, therefore, aimed at determining the pattern of prescription among diabetic patients relevant to current evidence and clinical guidelines. We tried to describe the socio-demographic characteristics of the diabetic patients getting prescription in Dhaka Medical College Hospital of Bangladesh and identify the pattern of prescriptions of hypoglycemic agents among them.

## METHODS

### Study design and population

It is a descriptive type of cross-sectional study conducted for a period of 3 months from 1 May to 31 July, 2015 among the diabetic patients, irrespective of age, sex and race, attending the outpatient department of diabetic clinic of Dhaka Medical College Hospital (DMCH) in Dhaka.

### Eligibility criteria & sampling technique

All diabetic patients who were willing to attend the study and were in optimal mental condition and receiving management for at least 6 months, irrespective of age, sex and race, attending the outdoor facility of diabetic clinic of DMCH. The patients who were disoriented or declining to participate in the study were excluded. Patients meeting the inclusion criteria & devoid of exclusion criteria were approached following purposive type of non-probability sampling technique, while they were waiting to see the physician. An interview was taken with a semi-structured case record form after getting informed written consent of the respondents. Demographic data as well as data on prescription were collected with this case record form.

### Sample size

At 5% margin of error and 95% confidence level, target sample size was determined using the following simple formula:^9^

$$n=\frac{Z^{2}P(1-P)}{m^{2}}$$

Where,

n= Sample size

Z= Z statistic for a level of confidence (At 95% Confidence level, Z= 1.96)

P= Expected prevalence or proportion

m= Margin of Error (in proportion of one; if 5%, m= 0.05)

$$n=\frac{{\left(1.96\right)}^{2}\times .09\times .91}{{\left(0.05\right)}^{2}}=126$$

In this study, due to time constraints, the researchers collected 105 samples.

Statistical methods Data were analysed, particularly mean, standard deviation and percentages were calculated and chi-square tests were done using SPSS v.22 while figures and tables were prepared using Microsoft Excel 2010. Categorical variables were presented in percentages.

## RESULTS

**Figure 1:**
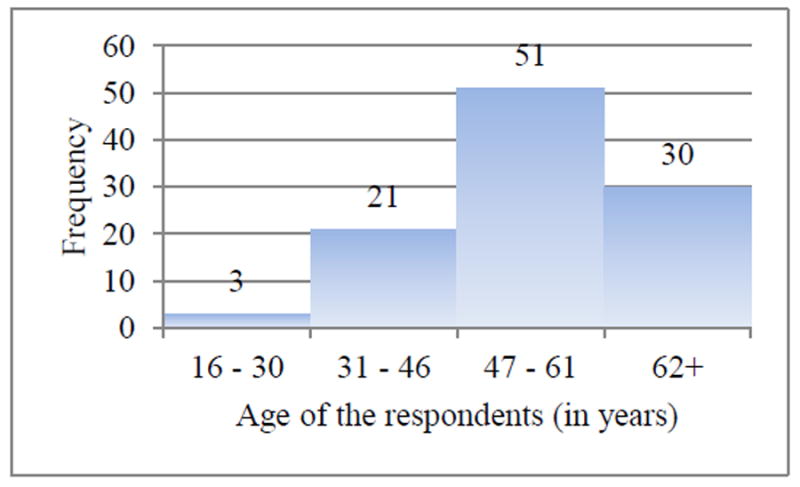
Distribution of age group of the respondents

**Figure 2:**
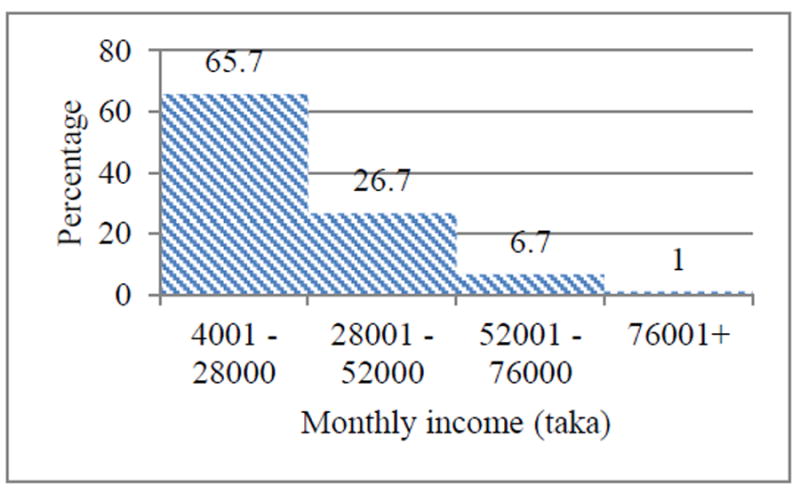
Distribution of monthly family income

**Figure 3:**
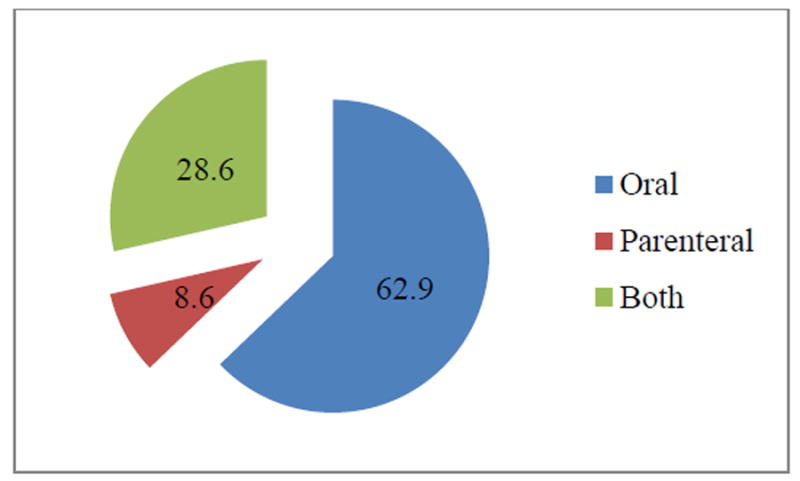
Distribution of dosage formulations

Among the total 105 respondents with mean age of 53.42 (SD±10.587) years, 40 (38.1%) were male and 65 (61.9%) female (Table 1) with male: female ratio of 0.62:1. Majority (69.5%) were from urban area and among them about 27.6% were male and 41.9% were female. The study revealed high prevalence (48.6%) of diabetes in 47-61 year age group (Figure 1). 39% were from primary level and only 6.7% were from post graduate group (Table 2). Majority (65.7%) were from monthly income of Tk. 4001-28000 group (Figure 2) with mean income of Tk. 26882.81 (SD± 14391.938). 81% of the respondents were Muslim and 17.1% Hindu (Table 3). 51.4% patients were diagnosed for less than 5 years while 17.1% were diagnosed beyond 10 years (Table 4). Majority (62.9%) of the patients were prescribed oral drugs singly and 8.6% injectable preparations alone (Figure 3). Among injectable drugs, majority (48.7%) had prescription of short acting insulin alone and 35.9% with mixed insulin preparation (Table 5). 66 patients had at least one co-morbidity and hypertension predominated (21.9%) the list (Table 6).

**Figure 4:**
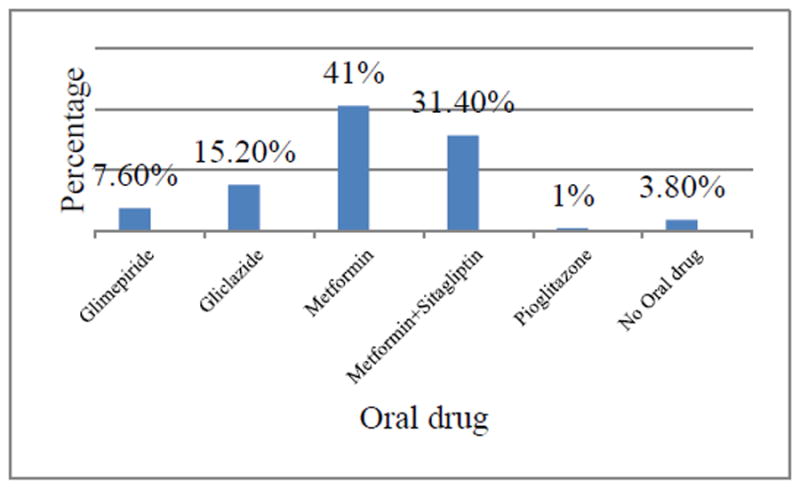
Distribution of prescribed oral anti-diabetic drugs

On an average, 5.62 (SD±3.163) drugs were prescribed per prescription where majority (54.2%) had 4-6 drugs (Table 7). 41% were prescribed Metformin alone and 31.4% Combination of Metformin and Sitagliptin (Figure 4). Majority (74.3%) of the drugs were from local manufacturers (Table 8).

## DISCUSSION

Out of 105 patients evaluated in the study, 40 (38.1%) were males and 65 (61.9%) were females. Females predominated in the study population which is in agreement with the result of a study in Taiwan, which indicated a possible epidemiological transition.^10^ This result does not corroborate with the findings of a cohort study conducted in the U.S. which also reported a male preponderance for DM.^11^

Majority (69.5%) of the respondents were from urban area, which corresponds to a previous study in Bangladesh.^12^ Similar findings have been reported previously in Bangladesh in another study (8% versus 4% in the two groups, respectively).^13^ Urban people have a more sedentary lifestyle or different dietary habits and are more likely to be overweight or obese, which may be the cause of DM to be more prevalent among them.^14^

The (Mean±SD) age of the patients was 53.42 (SD±10.587) years with a range between 20 and 76 years. It was higher than that reported in studies carried out in India (51.5 ± 12.3 years), lower to that reported in Hong Kong (56.5 ± 12.6 years).^15,16^

Maximum patients with DM were from the age group of 47 to 61 years followed by the age group of more than 62 and 31 to 46, which corresponds to a study in India.^17^ Greater prevalence in this age group may be due to change in life style, lack of physical exercise and stress.^17^ A total 66 patients suffered from comorbid conditions. Hypertension accounted for 34.3% of the total complications which was lower than in the study reported in Nepal.^7^ Our study findings are also similar to the study conducted by Arauz-Pacheco et al; in Texas medical center that hypertension is more common complication affecting 20-60% of people with diabetes.^18^

Figure 1 showing the distribution of age group of the respondents. Respondents’ age was divided into four groups. Majority of them (48.6%) were from 47-61 year age group. Their mean age was 53.42 (SD±10.587) years.

Figure 2 showing the distribution of monthly family income of the patients. Majority (65.7%) were from Tk. 4001-28000 group. About 26.7% were from Tk. 28001-52000 group. Respondent’s mean income was Tk. 26882.81 (SD± 14391.938).

Figure 3 showing the distribution of dosage formulations. 30 were prescribed with both the oral and injectable drugs concurrently among 105 respondents. Majority (62.9%) were prescribed oral drugs singly and 8.6% injectable preparations alone.

Figure 4 showing the distribution of the prescribed oral anti-diabetic drugs. It was found that among 105 respondents, 43 (41%) were prescribed Metformin alone and 31.4% Combination of Metformin and Sitagliptin. 3.8% patients were not prescribed any oral drug.

It was found that among 105 respondents, 43 (41%) were prescribed Metformin alone, which was the most and corraborates with other studies.^17,19^ Metformin and Sitagliptin was the most common drug in combination (31.4%). Majority (62.9%) was prescribed oral drugs singly, most probably due to the ease of administration.^17^ Only 39 (37.1%) were prescribed injectable hypoglycemic agents. Among them, majority (48.7%) were prescribed short acting insulin alone and 35.9% with mixed insulin preparation.

The duration of Diabetes Mellitus shows that, majority (51.4%) was diagnosed for within last 5 years. 17.1% were diagnosed beyond 10 years, indicating that patients present to the physicians more immediately after diagnosis.

About the dosage formulation of prescribed anti-diabetic agents, 30 were prescribed both oral and injectable drugs concurrently among 105 respondents. Majority (62.9%) were prescribed oral drugs singly and 8.6% injectable preparations alone.

Among the 105 respondents, the majority (54.2%) was prescribed 4-6 drugs and only 1.9% had maximum number of 17 drugs. The average number of drugs prescribed was 5.62 (SD±3.163) per prescription. In general, due to multiple co-morbidities, diabetic patients are at a greater risk of polypharmacy. Previous studies from a tertiary care hospital in Nepal identified lacunae in drug use pattern in the hospital.^20,21^

Local manufacturers had their majority (74.3%) of drugs in the prescription. Anti-diabetic drugs produced by both local and multinational manufacturers were in 23.8% prescriptions.

## CONCLUSION

Oral anti-diabetic drugs were advised in majority, which is easier method of drug administration. Physician’s preference was influenced or modified in some circumstances. However, this study found an underutilization of sulphonylureas and metformin-sulphonylurea combination, indicating the need for continuing medical education for physicians to more rationalize while choosing hypoglycemic agents. There is always a huge scope for improving prescription writing and treatment adherence by generic name. To reduce morbidity and mortality in diabetic patients, we must ensure optimum glycemic control not only by prescribing keeping in line with guidelines, but also by ensuring patients’ adherence to treatment plan.

### Limitations of the study

A larger study with probability sampling could not be possible due to time constraints & monsoon season. Certain information was recorded according to the statement of the respondents; as such the validity of the results was dependent on the validity of the statement given by the respondent, though data were recorded after careful judgment and calculation by the researchers.

### Recommendations

Depending upon the study findings, we can say that for better understanding of the pattern of prescription of hypoglycemic agents, larger study with probability sampling should be conducted. Health education should be given great importance in the mass media for diabetic patients irrespective of age, sex and area of residence, as it is one of the most effective tools for promotion of health. This will increase the adherence level for the patients. For the same purpose, awareness programs about diabetes control can be organized regularly.

## Figures and Tables

**Table 1:** Distribution of respondents by sex.

Sex	Number of respondents	Percentage
Male	40	38.1
Female	65	61.9
Total	105	100

**Table 2:** Distribution of the level of education of respondents.

Level of education	Number	Percentage
Below primary	10	9.5
Primary	41	39.0
Secondary	20	19.0
Higher secondary	19	18.1
Graduate	8	7.6
Post graduate	7	6.7
Total	105	100

**Table 3:** Distribution of religious status of the respondents.

Religion	Number	Percentage
Islam	85	81.0
Hinduism	18	17.1
Christianity	1	1.0
Buddhism	1	1.0
Total	105	100

**Table 4:** Distribution of duration of diabetes mellitus.

Duration of diabetes mellitus	Sex				Number	%
	Male		Female			
	Number	%	Number	%		
<5 years	22	21.0	32	30.5	54	51.4
5-10 years	13	12.4	20	19.0	33	31.4
>10 years	5	4.8	13	12.4	18	17.1
Total	40	38.1	65	61.9	105	100

**Table 5:** Distribution of the injectable anti-diabetic drugs prescribed (n=39).

Injectable drugs	Frequency	Percentage
Short acting insulin	19	48.7
Intermediate acting insulin	1	2.6
Mixed insulin	14	35.9
Short acting + Intermediate acting insulin	5	12.8
Total	39	100

**Table 6:** Distribution of co-morbidities associated with diabetes mellitus

Name of Co-morbidity	Frequency	Percentage
BEP	2	1.9
Cataract	2	1.9
Cataract, and Hypertension	2	1.9
Cataract, Hypertension and CKD	1	1.0
CKD	4	3.8
CKD with Hypertension	2	1.9
COPD	1	1.0
Hypertension	23	21.9
Hypertension, IHD	1	1.0
Hypertension, Obesity	1	1.0
Hypertension with PD	1	1.0
Hypertension with Stroke	2	1.9
Hypertension with Stroke and CKD	2	1.9
Hypertension with Stroke and PD	1	1.0
Hypothyroidism	2	1.9
IBS	1	1.0
IHD	2	1.9
LBP	1	1.0
Stroke with CKD	2	1.9
Osteoarthritis	6	5.7
Stroke	7	6.7
None	39	37.1
Total	105	100.0

BEP: Benign enlargement of prostate; CKD: Chronic kidney disease; COPD: Chronic obstructive pulmonary disease, IHD: Ischemic heart disease; PD: Parkinson’s disease; IBS: Irritable bowel syndrome; LBP: Low back pain.

**Table 7:** Distribution of the number of drugs prescribed for DM and associated co-morbidities according to sex.

Total number of drugs prescribed for everyday	Sex				Frequency	%
	Male		Female			
	Number	%	Number	%		
1-3	10	9.5	13	12.4	23	21.9
4-6	20	19.0	37	35.2	57	54.2
7-9	5	4.8	10	9.5	15	14.3
10-12	2	1.9	2	1.9	4	3.8
13-15	1	1.0	2	1.9	3	2.9
16-18	2	1.9	1	1.0	3	2.9
Total	40	38.1	65	61.9	105	100

**Table 8:** Distribution of dosage formulation according to the manufacturers of the anti-diabetic drugs.

Manufacturer of the anti-diabetic drugs	Dosage formulation of anti-diabetic agents prescribed						Frequency	%
	Oral		Parenteral		Both			
	Number	%	Number	%	Number	%		
Local	66	62.9	3	2.9	9	8.6	78	74.3
Multinational	0	0	2	1.9	0	0	2	1.9
Both	0	0	4	3.8	21	20.0	25	23.8
Total	66	62.9	9	8.6	30	28.6	105	100

X^2^ =143.377; df=4, p=0.000
